# Kimura Disease Presenting as Right Parotid Swelling and Neck Lymphadenopathy

**DOI:** 10.7759/cureus.18178

**Published:** 2021-09-21

**Authors:** Madan Shivakumar, Naveen Kumar Gaur, Sharmila Balaji, Oseen Shaikh, Uday Kumbhar

**Affiliations:** 1 Surgery, Jawaharlal Institute of Postgraduate Medical Education & Research, Puducherry, IND; 2 Pathology, Jawaharlal Institute of Postgraduate Medical Education & Research, Puducherry, IND

**Keywords:** immunoglobulin, neck swelling, lymphadenopathy, eosinophilia, kimura disease

## Abstract

Kimura disease is a rare chronic inflammatory disorder commonly affecting young males. We present a 35-year-old male who had right parotid region and neck swellings for two years. The patient underwent imaging studies, blood investigations, and fine-needle aspiration cytology diagnostic of the Kimura disease. The patient was screened for the presence of renal disorder. However, there was no evidence of kidney involvement. The patient was started on oral steroid therapy and advised for further follow-up.

## Introduction

Kimura Disease (KD) is a chronic inflammatory angiolymphatic proliferative disorder in young Asian males [1]. The etiology of KD is unknown and is characterized by a triad of painless subcutaneous masses in the head or neck region, blood and tissue eosinophilia, and markedly elevated serum immunoglobulin E (IgE) levels [2]. Although it may mimic a neoplastic process, the disease is a slowly progressive benign condition, which makes it difficult to make the correct diagnosis [3]. A high degree of suspicion and early diagnosis will help to avoid unnecessary invasive procedures on the patient. Here we report the case of a 35-year-old male with a rare presentation of involvement of lymph node, salivary gland, and subcutaneous tissue.

## Case presentation

A 35-year-old gentleman presented to us with right facial swelling, which started spontaneously as a small, indurated area and progressed in size gradually over two years. There was a history of vague discomfort in the area of swelling with an increase in the size of swelling in association with upper respiratory infections. There was no difficulty swallowing food, decreased saliva flow, pus discharge, or skin changes over the swelling. There were no features suggestive of facial nerve involvement or malignant nature such as weight loss, fever, or night sweating. No history of trauma or insect bite could be elicited. Other medical, surgical, and family histories were unremarkable.

**Figure 1:**
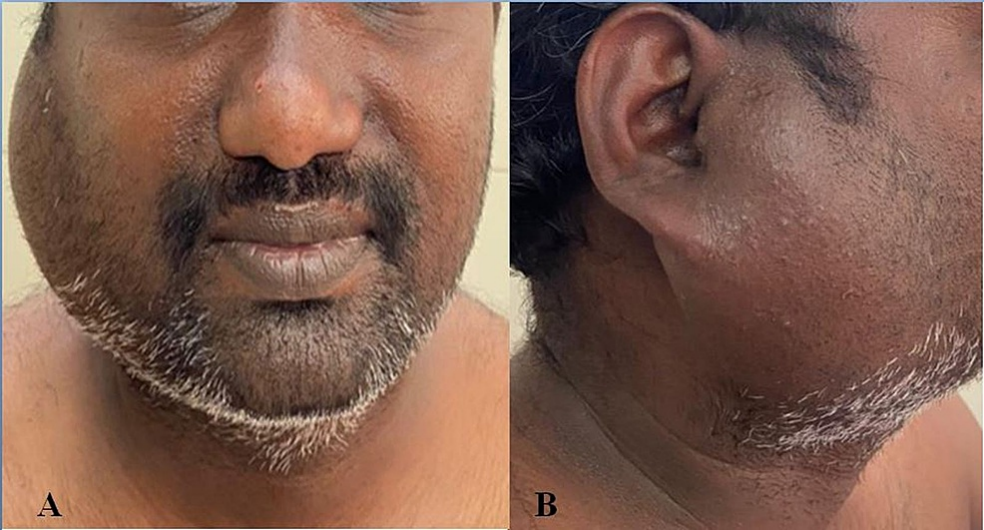
Clinical image showing enlarged left parotid gland; A: anterior view and B: lateral view.

Physical examination revealed a 7 cm x 6 cm well defined, firm, non-tender swelling with irregular borders extending from the right zygomatic arch to the mandible's lower edge, suggestive of an enlarged parotid gland (Figure 1).

The swelling was predominantly firm in consistency with scattered soft areas over it. The skin showed no warmth or redness and was stretched over the underlying swelling. Multiple enlarged right cervical lymph nodes were palpable. The skin over the neck lymph nodes was indurated. The left parotid gland was normal in size, and there was no left cervical lymphadenopathy. Systemic examination revealed normal findings.

Blood investigations showed hemoglobin of 14.4 g/dl, leucocytosis with an absolute eosinophil count of 6410 cells/mm^3^. Serum IgE level could not be determined. Other investigations like renal function tests and liver function tests were normal.

**Figure 2:**
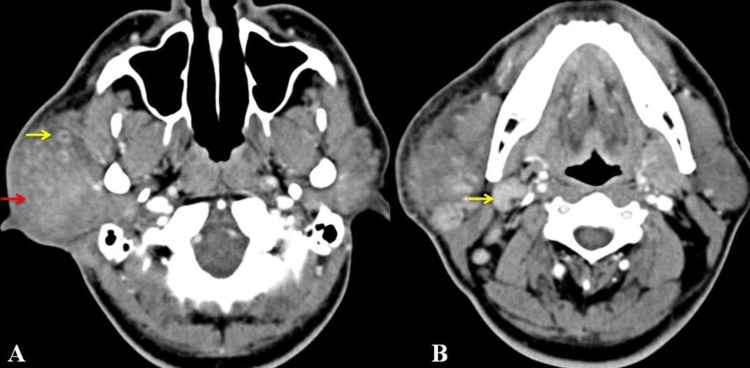
CT showing (A) enlarged left parotid gland (red arrow) with enhancing intraparotid lymph nodes yellow arrow and (B) enlarged neck lymph nodes (yellow arrow).

USG of the swelling showed a heterogeneous parotid gland with altered echotexture and multiple enlarged intraparotid lymph nodes with maintained fatty hilum. Few enlarged lymph nodes were noted in the right neck with maintained fatty hilum. The left parotid gland and other salivary glands were normal. CT showed a 7 cm x 6 cm x 6 cm diffusely enlarged parotid gland with patchy hyperenhancement with subcutaneous fat stranding around the parotid gland. There were enlarged hyper-enhancing cervical lymph nodes in the right side of the neck (Figure 2).

**Figure 3:**
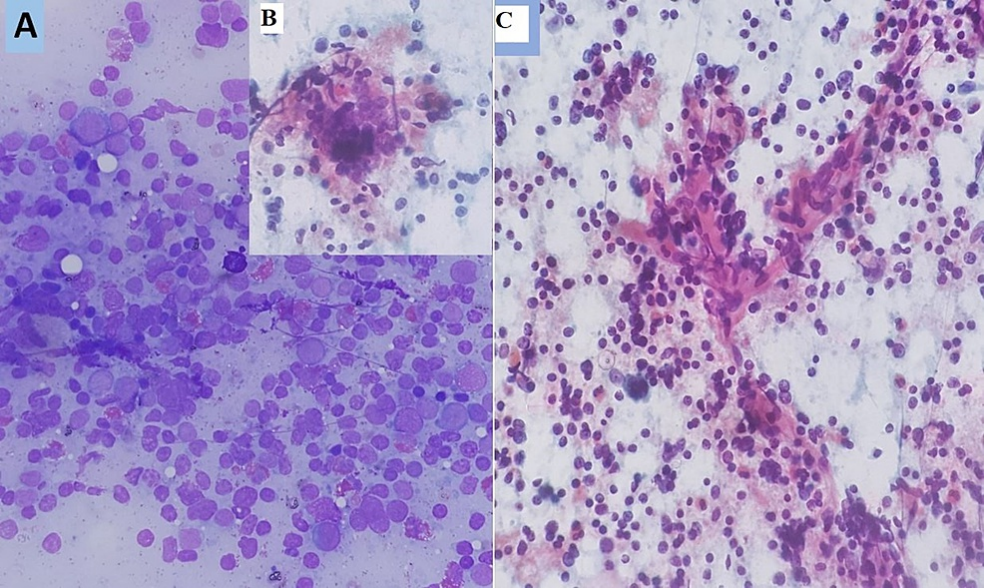
Cytological image showing (A) sheets of reactive lymphoid cells admixed with many eosinophils and an occasional mast cell, (B) Warthin-Finkeldy giant cell, and (C) a proliferating capillary lined by plump endothelial cells amidst reactive lymphoid cells mixed with eosinophils.

Fine needle aspiration cytology (FNAC) from the lesion showed sheets of the polymorphous population of reactive lymphoid cells with lymphohistiocytic aggregates, mast cells, tingible body macrophages, along with numerous eosinophils and a few Warthin-Finkeldey-like giant cells. No atypical cells, Reed-Sternberg cells, or granulomas were seen (Figure 3).

There was no evidence of proteinuria, hematuria, or any casts in the urine.

Our patient was started on oral steroid therapy. The patient was being followed for the next two months after the starting of the treatment. For the subsequent two-month follow-up, the patient had a mild decrease in the size of the swelling without any other complications.

## Discussion

KD is endemic to middle-aged Asian males in the second or third decades of life and is occasionally seen in non-Asian males [1]. Possible aetiologies include trauma, infection, an IgE-mediated hypersensitivity reaction, or an autoimmune process. However, the exact etiology of the disease in our patient could not be elicited.

KD presents predominantly as subcutaneous nodules in the head and neck, often unilateral with or without the involvement of salivary glands [2]. Other features include regional lymphadenopathy, peripheral eosinophilia, and elevated serum IgE [4]. Moreover, up to 60% of these patients exhibit renal involvement manifesting as extra membranous glomerulonephritis and nephrotic syndrome [3]. Most patients present with an indolent swelling around the auricular region, gradually increasing in size over the years. Our patient had predominant right parotid region swelling with multiple enlarged neck lymph nodes.

FNAC usually establishes the diagnosis of KD from the swelling, which generally shows significant numbers of eosinophils in a background of lymphoid cells with occasional fragments of collagenous tissue and Warthin-Finkeldey polykaryocytes [5]. In our case, FNAC from the right parotid swelling showed sheets of the polymorphous population of reactive lymphoid cells with lymph histiocytic aggregates, mast cells, and macrophages along with numerous eosinophils and a few Warthin-Finkeldey like giant cells.

Patients with KD have peripheral eosinophilia. It has been speculated that the degree of blood eosinophilia may be correlated with the size of the lesion, which might be used to measure the disease activity [6]. Our patient also had eosinophilia. The clinical features, the predominance of eosinophils, epithelioid endothelial proliferation, and the absence of atypical cells went against the likelihood of other neoplastic/infective lesions of the parotid gland.

USG is a valuable adjunct, usually showing enlarged solid lymph nodes with maintained hilar architecture and heterogeneous salivary glands along with increased hilar vascularity [7]. CT scan helps define the involvement of adjacent structures and shows intense enhancement of lymph nodes with the heterogeneous enhancement of salivary glands. It usually shows well-defined nodular masses in the subcutaneous tissue and lymphadenopathy, the most common site of involvement in the parotid region. MRI is helpful in the diagnosis. They are reported to have variable signal intensity on T1 and T2 weighted images. Even in the post-contrast images, enhancement may be very mild to intense [8]. This is due to the varying amount of fibrosis and vascular proliferation within the lesions. Our patient imaging features were consistent with KD. 

Asymptomatic cases are managed conservatively, and there has even been a case report of spontaneous resolution [9]. Symptomatic cases or cosmetic disfigurement warrants surgical excision, but the lesion tends to recur [10]. Systemically administered steroids like oral corticosteroids, cyclosporine, mycophenolate mofetil, leflunomide show promising effects on disease progression; however, withdrawal of steroids can often result in relapse. Radiation has been utilized for steroid-resistant lesions. Surgical excision is used only in localized KD. The overall prognosis of KD is good with no reports of malignant transformation, but renal involvement makes prognosis variable depending upon the form and severity of nephropathy [9,11]. Therefore, patients with KD should be monitored for disease recurrence and the development of renal disease.

## Conclusions

KD is a rare chronic inflammatory angiolymphatic proliferative disorder, usually present as neck swelling and parotid swelling. Initial presentation may masquerade like a malignant disease. Imaging studies give clues to the diagnosis. Definitive diagnosis is made by cytology or histopathology. Patients with KD are usually managed conservatively and have a good prognosis. 
